# 276. A Focused Screen Identifies Essential Genes Enabling Increased Expansion in Hematopoietic Stem Cells to Generate Human Neutrophils

**DOI:** 10.1093/ofid/ofad500.348

**Published:** 2023-11-27

**Authors:** Nathan E Jeffries, Kyle Timmer, Emma Yvanovich, Daniel Floyd, Michael Mansour, David B Sykes

**Affiliations:** Massachusetts General Hospital, Boston, Massachusetts; Massachusetts General Hospital, Boston, Massachusetts; Massachusetts General Hospital, Boston, Massachusetts; Massachusetts General Hospital, Boston, Massachusetts; Massachusetts General Hospital, Boston, Massachusetts; MGH, Boston, Massachusetts

## Abstract

**Background:**

Cellular therapies are of growing clinical interest, particularly for patients with deficiencies in white blood cell number related to chemotherapy or radiation exposure. Neutrophils are the predominant peripheral white blood cell, and their versatility and efficacy in fighting infections make them highly attractive for cellular therapy. As neutrophils have an inherent short lifespan and must be transfused in high numbers to be effective in protecting against infection, the development of neutrophil cellular therapy has been hindered by limitations in our ability to expand cells *ex vivo* from rare umbilical cord blood or bone marrow-derived hematopoietic stem cells (HSCs). Here we explore the use of a library of nine inducible genetic factors to augment the expansion of HSCs *in vitro* while maintaining their capacity to mature into functional neutrophils.

**Methods:**

Stable cell lines were generated via electroporation with a transposase system. Factors were screened simultaneously in random combination and subsequent combinations were selected based on successful ‘hits’ from initial screens. Cells were characterized via flow cytometry to confirm maintenance of a progenitor state through expression of the cell surface marker CD34 and differentiation into neutrophil-like cells, identified by surface expression of CD11b/CD15/CD66b. Clonal populations were analyzed via qPCR to determine which of the nine factors are linked to conditional immortality.

Methods & Experimental Outline

**Figure:**
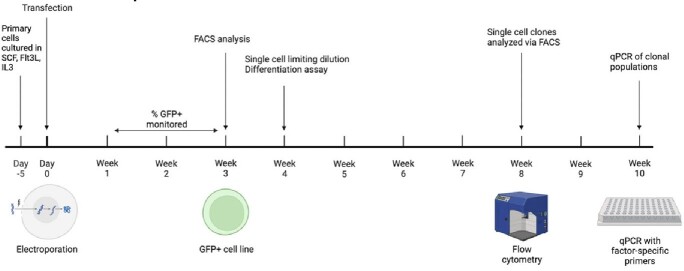

Timeline and methods for transfecting cells with our library and analyzing resultant cell lines.

**Results:**

Two out of three transfections using the nine-factor library yielded a stably transfected population which grew to comprise at least 75% of viable cells in culture after 3 weeks. CD34 positivity was maintained through 8 weeks of culture in some clones, and neutrophil-like cells were generated from one cell line after 5 weeks. Several clones divided continuously for 11 weeks. Specific factors associated with these phenotypes have been identified.

Flow Cytometric Analysis of Transfected Cells

**Figure:**
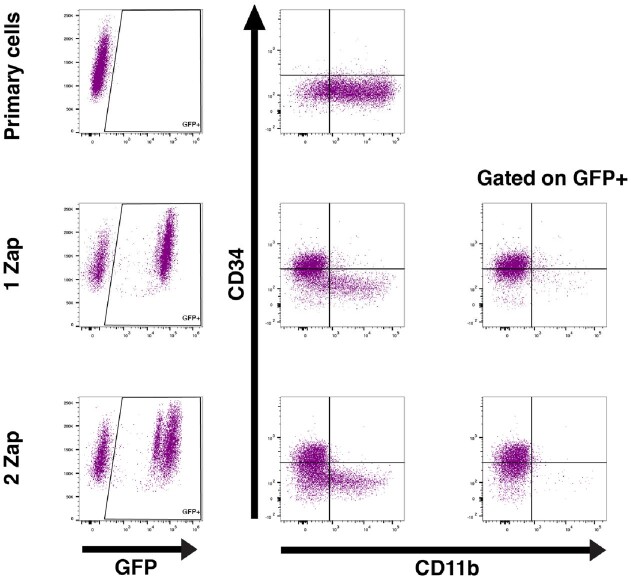

Cells were transfected via single (1 Zap) or double (2 Zap) electroporation then cultured for 3 weeks. Transfected cells express the immature surface marker CD34 more highly than untransfected primary cells and are lower for CD11b, a mature myeloid marker.

Flow Cytometric and Microscopic Analysis of Transfected Cells Ten Days After Induction of Differentiation

**Figure:**
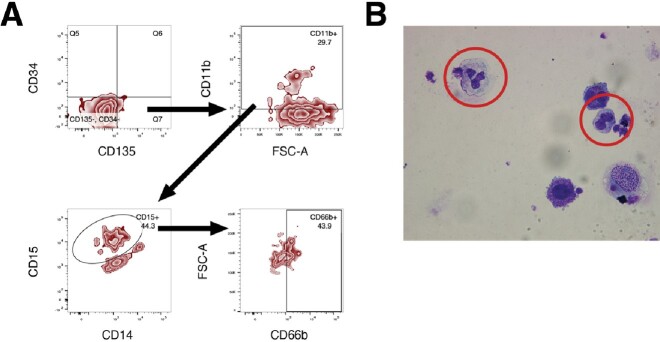

A ten-day differentiation assay was performed following 4 weeks in culture. A) Gating strategy used to identify a population of cells which is CD34(-), CD135(-), CD11b(+), CD15(+), and CD66b(+), and thus bear a neutrophil phenotype. B) Wright-Giemsa stain showing two cells with a neutrophil-like morphology.

**Conclusion:**

Enforced expression of a critical number of factors offers a developmental path for unlimited expansion of neutrophil precursors, making the generation of therapeutic neutrophil transfusions a realistic and feasible countermeasure for chemotherapy or radiation-induced neutropenia.

**Disclosures:**

**Nathan E. Jeffries, BA**, Safi Biotherapeutics: Salary **Michael Mansour, MD, PhD**, Thermofisher: Grant/Research Support

